# Supplementary material for: MetaRibo-Seq measures translation in microbiomes
Source: Nat Commun. 2020 Jun 29;11:3268. doi: 10.1038/s41467-020-17081-z (PMC7324362; doi:10.1038/s41467-020-17081-z)
Supplement: Supplementary file 10 — Supplementary Data 7 [file 41467_2020_17081_MOESM10_ESM.zip › File2/Confidence_VeryHigh_Taxonomy/333520_out.krona.html]

Javascript must be enabled to view this page.

members
magnitude
magnitudeUnassigned
count
unassigned
taxon
rank

333520\_out

29

29
2
superkingdom

976
phylum
29

29
200643
class

29
order
171549

171552
family
29

29
838
genus

2292365
species

SRS012849\_contig\_number\_17557
1

species
1263102
3

SRS016541\_contig\_number\_16862SRS049959\_contig\_number\_38381SRS144537\_contig\_number\_46037


SRS144537\_contig\_number\_47356
1
2293125
species

1

SRS077641\_contig\_number\_455
species
2292054

species
1262932
1

SRS144537\_contig\_number\_46559

165179
species

SRS011134\_contig\_number\_12336SRS013521\_contig\_number\_724SRS013687\_contig\_number\_contig-100\_5736.169287SRS015794\_contig\_number\_contig-100\_8576.66278SRS017307\_contig\_number\_15254SRS019808\_contig\_number\_18217SRS022609\_contig\_number\_37850SRS023914\_contig\_number\_contig-100\_2995.151593SRS045713\_contig\_number\_contig-100\_2700.121735SRS049896\_contig\_number\_9533SRS049959\_contig\_number\_15535SRS049995\_contig\_number\_13172SRS053356\_contig\_number\_contig-100\_1628.258915SRS054059\_contig\_number\_1404SRS074586\_contig\_number\_4588SRS078176\_contig\_number\_1447SRS1041031\_contig\_number\_21694SRS1041031\_contig\_number\_21694SRS1041116\_contig\_number\_828SRS1041140\_contig\_number\_14441SRS1041140\_contig\_number\_14441SRS104912\_contig\_number\_21010
22
